# An uncommon presentation and course of metastatic malignant melanoma: a case report

**DOI:** 10.1186/1752-1947-1-151

**Published:** 2007-11-26

**Authors:** Astrid Dalhaug, Adam Pawinski, Jan Norum, Carsten Nieder

**Affiliations:** 1 Radiation Oncology Unit, Department of Internal Medicine and Skin Diseases, Nordlandssykehuset HF, 8092 Bodø, Norway; 2 Department of Oncology, University Hospital of North Norway, Tromsø, Norway; 3 Institute of Clinical Medicine, Faculty of Medicine, University of Tromsø, Tromsø, Norway

## Abstract

Most patients with brain metastases from malignant melanoma are diagnosed after treatment for known extracranial metastases and have a poor outcome despite various local and systemic therapeutic approaches. Here we discuss an unusual case where a 45-year old patient presented with a brain metastasis as the first symptom of disease and where the presumed primary lesion later was found in the gastro-intestinal tract. Treatment consisted of sequential surgical removal of a total of 4 tumor sites (2 extracranially), whole-brain radiotherapy and two radiosurgery procedures within 13 months. Following her last treatment, the patient has now been in remission for 20 months. This case illustrates that some patients with multi-organ melanoma manifestations may benefit from the repeated use of effective local therapeutic approaches and may experience a quite favourable prognosis.

## Introduction

Malignant melanoma is next to lung cancer, the most frequent cause of brain metastasis [1]. These metastases usually develop late in the course of the disease. Only 7% of patients had brain metastases disclosed at the time of initial diagnosis [2]. It is uncommon that melanoma patients with brain metastases continue to have an occult primary tumor after initial thorough work-up and staging. In the study by Fife and coworkers, the figure was 14% [2]. The course of disease is typically characterised by rapid extracranial progression and short overall survival time despite various local and systemic treatment approaches. Here the authors discuss an unusual case where a patient presented with a brain metastasis as the first symptom of disease, a presumed primary in the gastro-intestinal tract and favourable survival and disease-control in the absence of any systemic therapy.

## Case presentation

**Figure 1:**
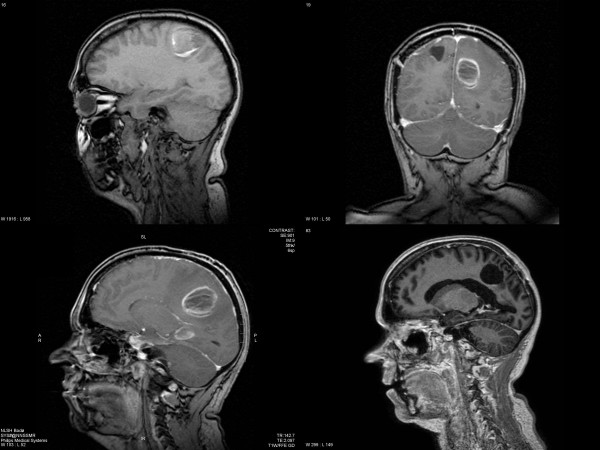
Magnetic resonance imaging scans of the brain. Upper left: the first lesion that led to diagnosis of metastatic melanoma. Lower left and upper right: 3 months after resection of the first lesion, 2 new metastases were diagnosed. Lower right: status 27 months after first diagnosis with residual changes after 2 radiosurgery treatments in the temporal lobe and a resection cavity in the parietal lobe.

The patient is a 45-year old caucasian female without serious previous disease or family history of cancer. In October 2004, she had noted a few days of hypesthesia in her left leg, followed by slight hemiparesis and a seizure resulting in hospitalisation. A magnetic resonance imaging (MRI) scan of the brain revealed a tumor in the right parietal lobe, presumably representing a glioma (Figure 1). In November 2004, a partial resection (because of the proximity to the motor cortex) was performed. The symptoms improved completely and the patient continued on anticonvulsant therapy. Histology demonstrated a metastatic tumor with pigmented cells and positive immunohistochemistry for S100, HMB45 and Vimentin.

**Figure 2:**
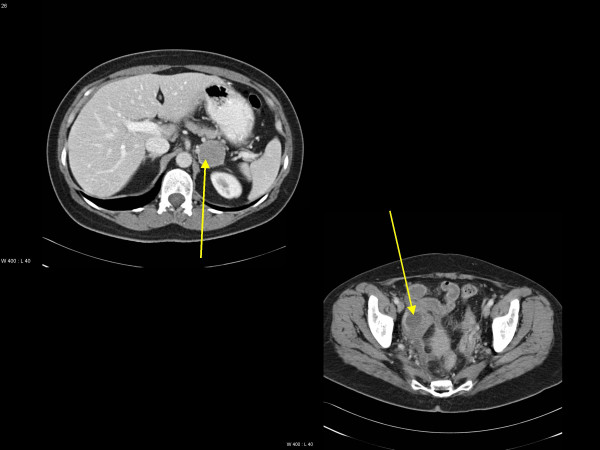
Computed tomography scans of the abdomen. Adrenal gland metastasis (upper scan) and lower abdominal mass resulting from inflammation around the melanoma in the vermiform appendix 4 months later (lower scan).

Staging including examinations of the eyes, head and neck mucosa and total skin, gynecological evaluation, bone scintigraphy and computed tomography (CT) scans showed an enlarged left adrenal gland as the only pathological finding (Figure 2). All routine blood tests and hormonal levels were within normal limits. The adrenal mass was removed completely by laparoscopic surgery and histology corresponded to that of the brain metastasis. Treatment proceeded with whole-brain radiotherapy (WBRT), 10 fractions of 3 Gy. In February 2005, the patient noted headaches and a decreasing general condition. A MRI scan disclosed two new brain metastases in the left parietal and temporal lobe, respectively (Figure 1). While the parietal tumor could be resected completely, the temporal lesion was treated with gamma-knife radiosurgery (RS). The peripheral minimum dose was 15 Gy. In March 2005, the patient developed abdominal symptoms and a CT scan showed a right abdominal mass presumably representing inflammation in and around the vermiform appendix and ovary (Figure 2). Surgery including ovarectomy and appendectomy was performed and the histology demonstrated again the same type of melanoma with all 3 positive markers as mentioned. The tumor was limited to the vermiform appendix without spread to peritoneum or lymph nodes and was judged to be removed completely.

After a symptom-free interval, routine MR evaluation in November 2005 disclosed tumor progression of the temporal lesion and a second gamma-knife procedure was performed. Since then, the patient returned to repeated follow-up examinations including MR and CT scans. The last one was performed in July 2007. As shown in Figure 1, there is a stable residual abnormality with contrast-enhancement in the temporal lobe after repeated gamma-knife treatments, possibly representing treatment effects rather than active tumor. No other potential signs of disease were detectable. The patient has a Karnofsky performance status (KPS) of 80% resulting from slight concentration and endurance problems. She has never received systemic treatment during the whole course of disease, although she initially was interested in participation in a clinical trial. However, all trials that were open in Norway at that time did explicitly exclude patients with brain metastases.

## Discussion

Neurological symptoms as the first sign of malignant melanoma are relatively uncommon, as is the inability to identify the primary tumor in patients with brain metastases from this disease [2]. The patient described here, had an adrenal mass that was detected shortly after diagnosis of the first brain lesion. Although primary adrenal melanoma has occasionally been described, the vast majority of adrenal lesions represent distant metastases [3]. It appears more likely that the primary tumor was located in the vermiform appendix, where it became symptomatic approximately five months after diagnosis of the disease. Primary melanoma arising from the mucosal epithelium of the gastrointestinal tract is also a rare entity and the differentiation between metastatic and primary tumors is very difficult [4]. However, no other lesion that might represent the primary was detectable in this patient.

Radiotherapy plays an important role in palliative treatment in this setting. Patients with a single brain metastasis managed with surgical resection plus WBRT have a 2-year survival rate of 20–25% [2]. Besides resection, prognostic factors included younger age, long disease-free interval and no concurrent extracranial metastases. In our patient, both salvage surgery and RS had to be performed relatively soon after WBRT. RS-reirradiation for local progression finally resulted in long-term control. It has recently been increasingly adopted that re-irradiation in primary and metastatic brain tumors might represent a valuable therapeutic option without unacceptable toxicity risk [5]. RS for melanoma brain metastases was reported to result in 1-year local control in 49% and overall survival in 25% of the patients, with survival being dependent on the score index for radiosurgery (SIR) [6]. The present patient belonged to the favourable SIR group (age ≤50 years, KPS >70%, no evidence of systemic disease at the time of RS, limited number of brain lesions and largest RS-treated lesion <13 ccm). The gamma-knife group from Pittsburgh described their results in 244 patients with melanoma brain metastases [7]. Median survival was 8 months and brain disease the cause of death in 40.5% of the patients. Those with controlled systemic disease, single brain metastasis and KPS 90–100% had better survival. A smaller recent series reported a median survival of 11 months and 2-year survival rate of 18% [8]. These authors emphasize that surgery or multiple RS procedures were associated with prolonged survival.

Throughout the melanoma literature, long-term survival after complete resection of metastatic disease has been reported repeatedly [9,10]. Despite new drugs, local responses after systemic treatment are infrequent, e.g., 10% in a recent report [11]. However, local response is significantly associated with longer survival [11]. Based on these facts and illustrated through the case discussed here, effective local therapeutic measures including, e.g., surgical resection and high-dose stereotactic radiotherapy, should be considered in patients with favourable prognostic factors and absence of rapid and synchronous multi-organ spread.

## Conclusion

This case illustrates that patients with multi-organ melanoma manifestations may benefit from the repeated use of effective local therapeutic approaches and may experience a quite favourable prognosis.

## Consent

Written informed consent was obtained from the patient for publication of this case report and any accompanying images. A copy of the written consent is available for review by the Editor-in-Chief of this journal.

## Competing interests

The author(s) declare that they have no competing interests.

## Authors' contributions

AD and AP treated the patient and collected the data. CN and JN drafted the manuscript. All authors read and approved the final manuscript.

## References

[B1] Barnholtz-Sloan JS Sloan AE Davis FG Vigneau FD Lai P Sawaya RE Incidence proportions of brain metastases in patients diagnosed (1973 to 2001) in the Metropolitan Detroit Cancer Surveillance System J Clin Oncol 2004 22 2865 2872 10.1200/JCO.2004.12.149 15254054

[B2] Fife KM Colman MH Stevens GN Firth IC Moon D Shannon KF Harman R Petersen-Schaefer K Zacest AC Besser M McCarthy WH Thompson JF Determinants of outcome in melanoma patients with cerebral metastases J Clin Oncol 2004 22 1293 1300 10.1200/JCO.2004.08.140 15051777

[B3] Rajaratnam A Waugh J Adrenal metastases of malignant melanoma: characteristic computed tomography appearances Australas Radiol 2005 49 325 329 10.1111/j.1440-1673.2005.01440.x 16026441

[B4] Schuchter LM Green R Fraker D Primary and metastatic diseases in malignant melanoma of the gastrointestinal tract Curr Opin Oncol 2000 12 181 185 10.1097/00001622-200003000-00014 10750731

[B5] Combs SE Gutwein S Thilmann C Debus J Schulz-Ertner D Reirradiation of recurrent WHO grade III astrocytomas using fractionated stereotactic radiotherapy (FSRT) Strahlenther Onkol 2005 181 768 773 10.1007/s00066-005-1415-6 16362786

[B6] Selek U Chang EL Hassenbusch SJ 3rd Shiu AS Lang FF Allen P Weinberg J Sawaya R Maor MH Stereotactic radiosurgical treatment in 103 patients for 153 cerebral melanoma metastases Int J Radiat Oncol Biol Phys 2004 59 1097 1106 10.1016/j.ijrobp.2003.12.037 15234044

[B7] Mathieu D Kondziolka D Cooper PB Flickinger JC Niranjan A Agarwala S Kirkwood J Lunsford LD Gamma knife radiosurgery in the management of malignant melanoma brain metastases Neurosurgery 2007 60 471 481 17327791 10.1227/01.NEU.0000255342.10780.52

[B8] Samlowski WE Watson GA Wang M Rao G Klimo P jr Boucher K Shrieve DC Jensen RL Multimodality treatment of melanoma brain metastases incorporating stereotactic radiosurgery Cancer 2007 109 1855 1862 10.1002/cncr.22605 17351953

[B9] Wong SL Coit DG Role of surgery in patients with stage IV melanoma Curr Opin Oncol 2004 16 155 160 10.1097/00001622-200403000-00013 15075909

[B10] Young SE Martinez SR Essner R The role of surgery in treatment of stage IV melanoma J Surg Oncol 2006 94 344 351 10.1002/jso.20303 16917867

[B11] Richtig E Ludwig R Kerl H Smolle J Organ- and treatment-specific local response rates to systemic and local treatment modalities in stage IV melanoma Br J Dermatol 2005 153 925 931 10.1111/j.1365-2133.2005.06796.x 16225601

